# Serum Stabilities of Short Tryptophan- and Arginine-Rich Antimicrobial Peptide Analogs

**DOI:** 10.1371/journal.pone.0012684

**Published:** 2010-09-10

**Authors:** Leonard T. Nguyen, Johnny K. Chau, Nicole A. Perry, Leonie de Boer, Sebastian A. J. Zaat, Hans J. Vogel

**Affiliations:** 1 Biochemistry Research Group, Department of Biological Sciences, University of Calgary, Calgary, Alberta, Canada; 2 Department of Medical Microbiology, Center of Infection and Immunity Amsterdam, Academic Medical Center, Amsterdam, The Netherlands; Johns Hopkins School of Medicine, United States of America

## Abstract

**Background:**

Several short antimicrobial peptides that are rich in tryptophan and arginine residues were designed with a series of simple modifications such as end capping and cyclization. The two sets of hexapeptides are based on the Trp- and Arg-rich primary sequences from the “antimicrobial centre” of bovine lactoferricin as well as an antimicrobial sequence obtained through the screening of a hexapeptide combinatorial library.

**Methodology/Principal Findings:**

HPLC, mass spectrometry and antimicrobial assays were carried out to explore the consequences of the modifications on the serum stability and microbicidal activity of the peptides. The results show that C-terminal amidation increases the antimicrobial activity but that it makes little difference to its proteolytic degradation in human serum. On the other hand, N-terminal acetylation decreases the peptide activities but significantly increases their protease resistance. Peptide cyclization of the hexameric peptides was found to be highly effective for both serum stability and antimicrobial activity. However the two cyclization strategies employed have different effects, with disulfide cyclization resulting in more active peptides while backbone cyclization results in more proteolytically stable peptides. However, the benefit of backbone cyclization did not extend to longer 11-mer peptides derived from the same region of lactoferricin. Mass spectrometry data support the serum stability assay results and allowed us to determine preferred proteolysis sites in the peptides. Furthermore, isothermal titration calorimetry experiments showed that the peptides all had weak interactions with albumin, the most abundant protein in human serum.

**Conclusions/Significance:**

Taken together, the results provide insight into the behavior of the peptides in human serum and will therefore aid in advancing antimicrobial peptide design towards systemic applications.

## Introduction

Cationic antimicrobial peptides are an essential component of the defense systems of organisms throughout nature and they offer protection from invading pathogens [1]. Over the last two decades, research has blossomed in this field with the ultimate aim of developing antimicrobial peptides into viable alternatives for antibiotics that can act on multidrug-resistant bacteria, which are becoming increasingly prevalent [2]. The advantage of antimicrobial peptides is the generality of their mechanism of action, which involves either compromising the bacterial membrane integrity or disrupting essential components inside the cells [3], [4]. This differs from the specific receptors targeted by conventional antibiotics which allow the pathogenic bacteria to develop resistance more rapidly. Furthermore, antimicrobial peptides are fast-acting and biodegradable, which alleviates the current concern over residual antibiotics in the environment. In addition to their direct microbicidal activities, these host defense peptides are particularly attractive because of the multiple activities that are associated with many members of this family. These include the regulation of the innate and adaptive immune systems, inflammation and wound healing [1], [2], and additional anti-infective activities such as being antifungal, antiviral, antiparasitic or anticancerous [5]–[8].

The development of antimicrobial peptides for clinical use has thus far been limited to topical applications [2]. Although they could in principle be used systemically, proteolytic degradation and efficient peptide clearance pose as significant obstacles in the dynamic circulatory system. This problem is compounded by the relatively high cost of production of synthetic peptides, thereby making shorter peptide candidates more desirable [9], [10]. Many design strategies have been employed to date to optimize antimicrobial activity and selectivity. Through such peptide engineering, the peptides can also be made less susceptible to serum proteases. For example, modifications with unnatural amino acids such as D-amino acids [9], derivatives with unnatural side chains [11], and β-amino acids [12] have been shown to improve the stability and activity in several cases. Cationic compounds with stable steroid backbones resembling antimicrobial peptides have also been studied [13]. Cyclization can also be a viable strategy to constrain the peptide structure and thereby enhance antimicrobial activity. For example, this can be achieved by joining the terminal ends of a peptide by a disulfide bridge [14]–[17]. This approach has been used for an analogue of indolicidin, an antimicrobial peptide isolated from bovine neutrophils, where a disulfide-bridged peptide was more resistant to trypsin degradation than its linear counterpart [15]. Head-to-tail backbone cyclizationis another effective modification to constrain the peptide structure [18]–[20], and it has the added advantage of eliminating any susceptibility to exoproteases [21]. In fact, nature produces a number of cyclic antimicrobial peptides itself such as rhesus theta-defensin [22], microcin J25 [23] and daptomycin [24].

Antimicrobial peptide administration into the circulatory system raises a number of concerns regarding their activity, as they may not be as effective as observed in *in vitro* assays. Many peptides can be inactivated by physiological salt concentrations, as exemplified by the β-defensins [25], [26], magainin [27] and LL-37 [28]. However, several peptides have salt-insensitive activities including clavinin [27], protegrin [29] and tachyplesin [30]. In fact, some synthetic α-helical peptides have been engineered to be salt-resistant by fine-tuning the amphipathicity and stability of the α-helix [31], [32]. Possible interactions with serum proteins may also impede activity, particularly when the peptides bind to abundant proteins in blood plasma such as human serum albumin (HSA). Recently, a set of cationic antimicrobial tripeptides were found to interact with bovine serum albumin (BSA) with micromolar affinities, and their antimicrobial activities were reduced five-fold or more in BSA-supplemented assays [33]. This effect was also observed with human β-defensin-3 where the addition of HSA reduced its activity against *Acinetobacter baumannii* and abolished its activity against *Staphylococcus aureus* [25]. Despite these obstacles, the successful use of some antimicrobial peptides for treating specific infections has been demonstrated in mouse models [34]–[37]. Moreover, injected peptide drugs are now becoming more commonly used, for example in the treatment of HIV and chronic pain [38], [39].

In this study, the serum stabilities of two sets of hexameric antimicrobial peptide sequences were tested. The first group of peptides is based on the primary sequence RRWQWR. This sequence has been referred to as the antimicrobial center of bovine lactoferricin (LfcinB) [40]. LfcinB itself is an antimicrobial 25-residue peptide that is released from the cationic N-terminal region of bovine lactoferrin upon pepsin digestion in the gastrointestinal tract [8]. The sequence of the second group of peptides, RRWWRF, was identified through screening of a synthetic combinatorial hexapeptide library and is hereafter named Combi in this study [41]. These two sequences highlight the importance of tryptophan and arginine residues in short antimicrobial peptides. The Arg side chains provide positive charges as well as hydrogen bonding capabilities to attract the peptides to the negatively charged phospholipid headgroups of the bacterial membranes, and the indole side chains of Trp have a preference for the interfacial region of lipid bilayers [3], [42], [43]. While membrane interactions are important for these peptides [44], confocal microscopy data obtained for the Combi peptide and several analogs show that they can rapidly become localized in the cytoplasm of bacterial cells, which suggests that they act on intracellular targets and that they are not strongly membrane-active [9], [45].

The variations within the group of peptides studied here involve well established simple peptide modifications. These include acetylation and/or amidation of the N- and C-termini, respectively, which allows us to examine how neutralization of the terminal charges influences their susceptibility towards exopeptidases. Carboxyamidation has been a popular modification in synthetic and naturally occurring antimicrobial peptides because of the removal of the carboxyterminal negative charge, which normally gives rise to an increased activity [46], [47]. In some cases, this specific modification may also lead to the stabilization of the peptide structure [48]. The LfcinB-derived hexamer was originally studied with a free N-terminus and an amidated C-terminus, and the Combi peptide was originally studied with both of these modifications. The hexapeptides were also cyclized by both disulfide crosslinking and head-to-tail linking of the peptide backbone for comparison [21]. The backbone-cyclized version of the Combi peptide has earlier been shown to possess increased antimicrobial potency [18], however its stability against degradation has not yet been studied. To complement our studies with the hexameric peptides, we also studied linear and cyclic derivatives incorporating eleven residues from LfcinB [14] to explore the effect of peptide length on peptide stability in serum. The amino acid sequences of the peptide variants studied here are listed in Table 1. To determine their stability in human serum, peptide amounts were quantified by HPLC and mass spectrometry was used to identify the fragments produced. The antimicrobial activity of all the modified peptides was also determined so that peptides with both optimal stability and activity could be identified. Finally, the interactions between these peptides and human and bovine serum albumin were investigated by isothermal titration calorimetry (ITC) to further characterize their behavior in the bloodstream.

**Table 1:** Sequences and net charges of the short LfcinB and Combi peptides.

Peptide	Sequence a	Net charge at pH 7.0
Lfc1	RRWQWR	+3
Lfc2	RRWQWR-NH2	+4
Lfc3	CH3CO-RRWQWR	+2
Lfc4	CH3CO-RRWQWR-NH2	+3
Lfc5	-RRWQWR-	+3
Lfc6	CRRWQWRC-NH2	+4
Lfc7	RRWQWRMKKLG	+5
Lfc8	-RRWQWRMKKLG-	+5
Lfc9	CRRWQWRMKKLGC-NH2	+6
		
Com1	RRWWRF	+3
Com2	RRWWRF-NH2	+4
Com3	CH3CO-RRWWRF	+2
Com4	CH3CO-RRWWRF-NH2	+3
Com5	-RRWWRF-	+3
Com6	CRRWWRFC-NH2	+4
Com7	CH3CO-CRRWWRFC-NH2	+3

a Hyphens around certain sequences represent head-to-tail backbone cyclization; underlined sequences denote peptides with disulfide bridging between the terminal Cys residues.

## Results

### Stabilities in human serum

**Figure 1:**
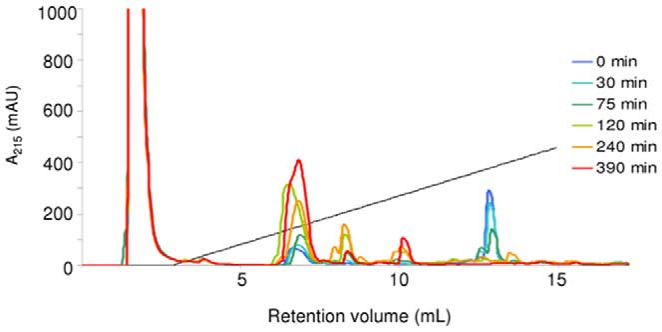
An example set of HPLC chromatograms from various time points of Lfc9 incubated in 25% human male serum at 37°C, showing the degradation of the intact peptide (∼13 mL ret. vol.) and appearance of its partially digested products (5–11 mL ret. vol.).

**Figure 2:**
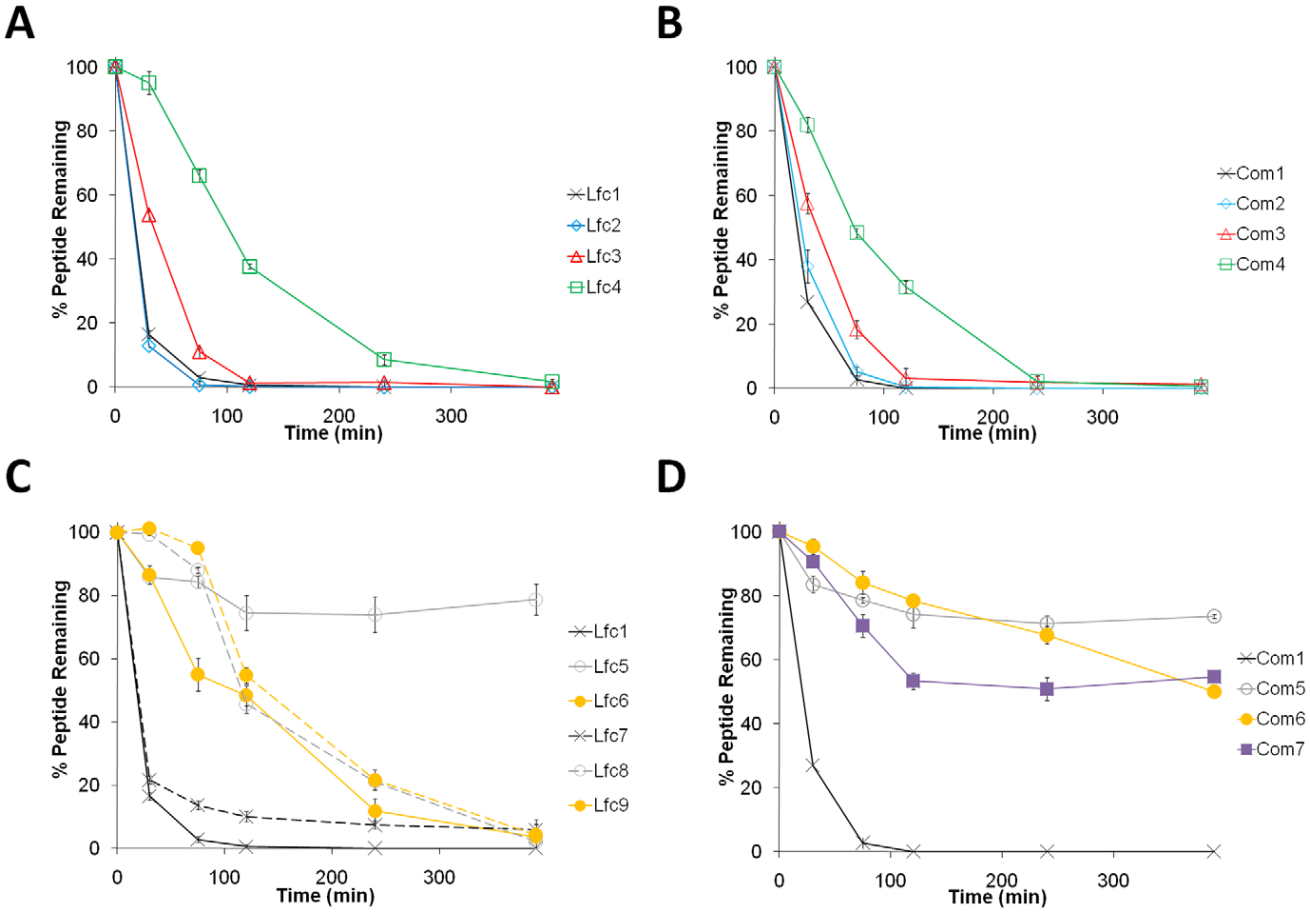
Serum stability profiles of the LfcinB- and Combi-based peptides. Relative peptide concentrations were determined by integration of the A215 peaks from RP-HPLC chromatograms. A) Linear LfcinB hexamers showing individual and combined effects of C-terminal amidation and N-terminal acetylation, B) Linear Combi hexamers with end caps C) Linear and cyclized LfcinB peptides comparing the end-to-end disulfide bridging and head-to-tail backbone cyclization strategies to different sized peptides, and D) cyclized Combi peptides. Assays were performed in triplicate.

To investigate the effects of the modifications on proteolytic susceptibility, the disappearance of the intact peptides incubated in diluted human serum at 37°C was followed by RP-HPLC. A sample set of chromatograms is shown for Lfc9 in Fig. 1. Incubations were normally done for 9 h and, in some cases, longer. Overall results in Fig. 2 display only the course of degradation up to 6.5 h to highlight the differences in the profiles of the rapidly degraded peptides.

The linear hexamers show little difference between LfcinB- and Combi-based primary sequences (Fig. 2A and B). The peptides with free N- and C-termini, Lfc1 and Com1, are degraded quickly with half-lives of less than 0.5 h and are completely gone after 2 h. Lfc2 and Com2 show very similar profiles for the non-modified peptides, suggesting that C-terminal amidation does not protect against carboxypeptidase activity in human serum. The N-acetylated hexamers seem to confer some stability against aminopeptidases, as both Lfc3 and Com3 have half-lives of about 1 h. However both peptides are still almost fully degraded after 2 h. Protecting both ends of the peptides gives them a greater stability beyond the additive contributions of the individual modifications. The capped hexamers have half-lives of ∼1.5 h with Lfc4 being notably more resistant than Com4.

In contrast, cyclization of the short peptides generally brings about significant stabilization against serum proteases (Fig. 2C and D). Lfc5 and Com5 are both remarkably resilient and more than 70% of these peptides remain intact after 6.5 h. 24 h serum incubations were also carried out for these peptides and their levels at this time period were ∼50%. The disulfide bonded short peptides are more susceptible to degradation. In fact, Lfc6 has a degradation profile similar to that of Lfc4. Com6 and Com7 are more resistant with ∼50% intact peptide left after an incubation time of 6.5 h. Beyond this, the 9 h time points continue to show the gradual degradation. Com6 is initially degraded more slowly than Com7, which is unexpected given the results of their linear counterparts. Nevertheless, their levels stabilized after 6.5 h.

The linear 11-mer LfcinB peptide, Lfc7, is slightly less degraded in serum compared to the hexameric Lfc1. The cyclized analogues are considerably more resistant to the serum proteases than Lfc7, however their levels all approach 0% intact peptide remaining after 6.5 h. The stability observed for the hexameric Lfc5 does not translate to the longer 11-residue cyclic peptide, Lfc8. Instead, the latter peptide's degradation profile is similar to that of Lfc9.

### Mass spectrometry characterization of intermediate fragments

To examine the cleavage patterns of the peptides in serum, aliquots of each incubation mixture were taken at the 75 min time point to be analyzed by MALDI mass spectrometry. The peptides which had observable intermediate fragments are reported in Table 2 with cleavage product identification. For the hexamers with free termini or an amidated C-terminus, no intermediates were recovered. This is probably due to the fast degradation of these peptides in the diluted serum. The acetylated hexamers do have identifiable fragments which correspond to the loss of the last two or three residues. The same fragments are recovered in the peptides that also have amidated C-termini, which indicates that the loss of the last two or three residues is not due to carboxypeptidase activity. Instead, there are clear recognition sites for endoproteases. No intermediates were recovered from incubations with the cyclic hexamers except for a nicked Lfc5. This may be due to the additional backbone rigidity of these peptides that would decrease possible interactions with proteases. The intermediate fragments recovered from Lfc8 help to explain its lowered stability. A notable cleavage site is present preceding either the last or the penultimate residue of the sequence when the peptide backbone extends well beyond those residues as in Lfc8, whereas with Lfc7, there may not be enough of a peptide extension to have proper interactions with the same endopeptidase.

**Table 2:** Fragments identified by MALDI-MS from 75 min peptide incubations in diluted human serum at 37°C a.

Original Peptide	Fragment mass (Da)	Assignment
Lfc3	687.3	CH_3_CO-RRWQ
	559.3	CH_3_CO-RRW
Lfc4	687.4	CH_3_CO-RRWQ
	559.3	CH_3_CO-RRW
Lfc5	985.1	Single Cut b
Lfc7	960.3	RWQWRM
	806.2	WQWRM
Lfc8	1544.8	Single Cut b
	943.5	KKLGRRW/KLGRRWQ c
	815.5	KLGRRW/LGRRWQ c
Com2	745.3	CH_3_CO-RRWW
Com4	745.4	CH_3_CO-RRWW
	559.3	CH_3_CO-RRW

a Only peptides with observed intermediate fragments are listed here.

b These fragments correspond to backbone cyclized peptides with one hydrolyzed peptide bond.

c Unambiguous assignment of these fragments was not possible.

### Interactions with serum albumins by ITC

The potential for interactions between various peptides and bovine and human serum albumin (BSA and HSA) were studied by microcalorimetry. The results were similar for all peptides regardless of primary sequence, cyclization, length and source of albumin. Examples of two ITC profiles can be found in Fig. S1. Two-site modeling was used for data fitting in the Origin software, suggesting two different modes of interaction. Overall, the interactions between these peptides and the serum albumins are weak, with the strongest dissociation constants (*K_D_*) in the low millimolar range.

### Antimicrobial and hemolytic activities

All the peptides were assayed for bactericidal activity against *B. subtilis*, *S. aureus* and *E. coli*, in sodium phosphate buffer supplemented with tryptic soy broth (Table 3). The concentrations killing 99.9% of the inocula (LD_99.9_) are in general agreement with antimicrobial minimum inhibitory concentration (MIC) data for similar peptides that have been used in previous studies [18], [40], [41], [44], although there are small discrepancies which can be attributed to differences in assay conditions and bacterial strains.

**Table 3:** Antimicrobial activities of the short LfcinB and Combi peptides.

Peptide	99.9% Lethal Dose Concentration (LD99.9) (µM)		
	*B. subtilis*	*S. aureus*	*E. coli*
Lfc1	7.5	30	60
Lfc2	1.9	15	15
Lfc3	60	>120	120
Lfc4	3.8	60	15
Lfc5	1.9	7.5	7.5
Lfc6	0.9	3.8	15
Lfc7	0.9	7.5	15
Lfc8	≤0.45	15	3.8
Lfc9	≤0.45	≤0.45	1.9
Com1	1.9	7.5	60
Com2	0.9	30	30
Com3	7.5	30	120
Com4	0.9	7.5	7.5
Com5	≤0.45	7.5	30
Com6	0.9	0.9	3.8
Com7	≤0.45	1.9	30

In general, all the modifications have significant effects on peptide microbicidal activity. The strains of the Gram positive species, *S. aureus* and especially *B. subtilis*, are more susceptible to killing than the Gram negative *E. coli* strain. The Combi-based peptides are generally more potent as antimicrobials compared to the corresponding LfcinB-based hexamers. This is perhaps not surprising given that the primary sequence of the former peptide was obtained after extensive screening of a combinatorial library.

Com3 and especially Lfc3 are the least cationic peptides and as a result they display the lowest activity in their respective series. In fact, their activities against *E. coli* are negligible. On the other hand, when the negatively charged C-terminus is neutralized by amidation as with Lfc2 and Com2, there is an increase in potency compared to Lfc1 and Com1. However, this advantage is not all-encompassing as seen by the reduced activity of Com2 against *S. aureus* compared to Com1. The activities of the peptides with both end caps do not simply reflect an additive combination of these opposing influences. Against *B. subtilis* and *E. coli*, Lfc4 shows similar activities compared to Lfc2. However, against *S. aureus*, the effect of N-terminal acetylation is stronger and the peptide is less active than Lfc1. On the other hand, the acetylated N-terminus in Com4 is beneficial and has a synergistic effect with the C-terminal amidation so that its activity against *S. aureus* and *E. coli* is improved compared to Com2.

The cyclized peptides are considerably more active than their linear counterparts. While cyclization by backbone peptide linkage improves the potency of the short antimicrobial peptides, disulfide bridging between two added terminal disulfides appears to be even better. This may in part be due to the amidated C-termini of the disulfide bridged peptides that was chosen to be included in our design of optimal antimicrobial peptides to maximize the net charge. Com7 was also designed as a complement to Com6 due to the greater activity of its linear counterpart. The modification of both termini in Com7 gives mixed results compared to Com6 with an improvement in activity against *B. subtilis*, the same level of activity against *S. aureus*, and being less effective against *E. coli*.

The 11-mer LfcinB-derived peptides show greater activities than the hexamer peptides. This reflects the higher number of positively charged and hydrophobic side chains in the sequence of the longer peptides. Lfc9 and Lfc8 are once again very active compared to their linear version, Lfc7, with the exception of Lfc8 losing some activity against *S. aureus*.

The peptides were also tested for cytotoxicity with hemolytic assays. None of the peptides are hemolytic and therefore, they should be associated with high therapeutic values.

### Antimicrobial activities in the presence of serum

In order to be able to make a direct comparison with our serum stability measurements, a few peptides were selected to be tested for antimicrobial activity in 25% human serum (Table 4). The *B. subtilis* and *S. aureus* strains were initially chosen for these assays because of the high activities associated, however the *B. subtilis* strain did not grow in serum conditions. All LD_99.9_ values are higher compared to the initial assays in 10 mM sodium phosphate buffer. Contributing factors to this general loss in activity could be the increased ionic concentration, interactions with serum components such as albumin or other proteins, and peptide degradation. Both hexamers with unprotected termini, Lfc1 and Com1, are no longer active while the cyclized short peptides remained fairly antimicrobial. In human serum, Lfc5 and Lfc6 are equally anti-staphylococcal. This reflects a greater loss of activity for Lfc6, but also coincides with the lower serum stability that this peptide has compared to Lfc5 (Fig. 2C). The activities of the cyclized Com peptides were all lowered equally with a 16-fold increase in LD_99.9_ values compared to activities in serum-excluded media and this agrees with their similar stability profiles (Fig. 2D).

**Table 4:** Anti-*S. aureus* activities of selected peptides in 25% human serum.

Peptide	LD_99.9_
Lfc1	>120
Lfc5	60
Lfc6	60
Com1	>120
Com5	30
Com6	15
Com7	30

## Discussion

Current antimicrobial peptide research generally focuses on the discovery and development of new peptides with superior activity. However, little attention has been paid so far to characterizing their behavior in their intended clinical environment. Important pharmacokinetic aspects to consider about the peptide drug candidates include their *in vivo* activity, cytotoxicity, circulation and targeting, degradation as well as clearance, and side effects. It has been reported that the *in vivo* stability of peptides in blood is well modeled by *in vitro* stability in serum or plasma [49].

The hexameric peptides studied here are rich in Trp- and Arg- residues, which are known to have high antimicrobial activity and low hemolytic activity [3], [18], [44]. Confocal microscopy studies and fluorescent dye leakage assays show that, like buforin and several other peptides [50], these short linear peptides localize to the cytoplasm of bacterial cells and are not highly disruptive to model membranes resembling bacterial membranes. Therefore, an intracellular mechanism of action is at least partially responsible for their antimicrobial activity, presumably by binding to and interfering with RNA and DNA synthesis [9], [14], [45]. However, peptide/membrane interactions remain important as they can modulate selectivity even when a peptide is not membrane permeabilizing [51]. The originally studied Lfc2 and Com4 peptides were found to be unstructured in aqueous solution and they become ordered and adopt a well-defined conformation when bound to lipid membranes [42], [43]. The micelle-bound structures of these peptides as determined by NMR spectroscopy are amphipathic with a positively charged side consisting of three comparatively flexible Arg side chains and a hydrophobic side consisting of the aromatic residues that are embedded in the interfacial region of the membrane [52].

The antimicrobial data reinforces the notion that C-terminal amidation improves peptide activity, most intuitively by removing a negative charge. In terms of proteolytic susceptibility, the protection of the C-terminus alone has a negligible effect. Supporting mass spectrometry data detect only peptide fragments that had two or three residues missing from the C-terminal ends of the peptides after 75 min serum incubation. The absence of a fragment that is missing only one residue suggests that serum endopeptidases have to cleave near the middle of the peptides before a carboxypeptidase will utilize the peptide as a substrate.

N-terminal acetylation decreases antimicrobial activity although there may be some cases where the combination of both modifications is even better for peptide activity as seen with Com4. This potentially beneficial modification is not employed as often as C-terminal amidation alone. The serum stability assays and subsequent mass spectrometry data indicate that the terminal end caps are somewhat advantageous for proteolytic stability as well. N-terminal acetylation confers notable resistance against serum aminopeptidases. There is even greater improvement in combination with carboxyamidation. This suggests that these modifications serve not only to protect against exopeptidases, but also endow the peptides with greater structural stability so that they are more resistant to endoproteases. However, the linear hexamers remain more susceptible to degradation than their cyclic counterparts.

Peptide cyclization greatly improves both the serum stability and microbicidal activity of the peptides. NMR studies of Com5 have shown that the peptide forms a reasonably defined structure in aqueous solution, but the micelle-bound peptides have increasingly similar structures, particularly converging around the aromatic residues [18]. The Trp and Phe side chains point toward one side of the backbone ring and form a better defined hydrophobic face that is stabilized by interactions with the micelle. Between the two cyclization strategies, disulfide bonding by terminal cystines is clearly more advantageous for antimicrobial activity than backbone linkage. In the case of Lfc5 however, its higher serum stability compensated for the lower activity so that its activity in serum is close to that of Lfc6. Due to the close stability profiles of the cyclic Com peptides, the disulfide-bonded peptides remain the most potent peptides in human serum, particularly Com6.

The longer LfcinB-derived peptides have the same benefits from cyclization for antimicrobial activity. This trend agrees with other peptides of similar sizes such as indolicidin and pyrrhocoricin [15], [19]. The previously determined micelle-bound NMR structures of Lfc7 and Lfc9 show that the cyclized peptide is more amphipathic, with its hydrophobic side chains embedded deeper into the membrane to give rise to better activity than the linear peptide [14]. With the full length LfcinB however, the native disulfide bond that connects the ends of the peptide does not have a significant functional role, as seen by the similar activities between the oxidized and reduced forms of the peptide. While the disulfide is essential for stabilizing the β-sheet structure of LfcinB in aqueous solution, the presence of the shorter “active center” appears to govern the antimicrobial activity.

The improved structural rigidity of the backbone-cyclized 11-mer does not translate into the remarkable protease stability that is observed with the related backbone-cyclized hexameric peptide. Thus, there is a greater advantage in constraining shorter peptides so that their backbones are less inviting substrates for serum proteases. Evidently, the shorter length of Lfc5 presents less possible active sites for endoproteases than Lfc8. This is supported by the observation that the major fragments from the Lfc8 incubation with human serum result from cleavage sites outside of the RRWQWR sequence. Interestingly, the Lfc8 fragments are completely different from the Lfc7 intermediates. This indicates that the linear and cyclic peptides differ in conformation so that the sites most vulnerable to proteolysis are not common to both peptides. In the case of the 20-residue pyrrhocoricin, a cyclic derivative incorporating a 9-residue linker was observed to be even less stable in human serum than the native peptide [19].

Another major serum component that may affect the action of these antimicrobial peptides is serum albumin. In this work, ITC experiments were carried out to study the thermodynamics of BSA and HSA binding to the Trp- and Arg-rich peptides. All of the ITC results obtained were comparable to each other, with millimolar affinities calculated from the curve fittings. These weak interactions likely have little effect on peptide stabilities or activities in the serum, although they cannot be ruled out as a contributing factor because of the high concentrations of HSA in human serum. Comparatively, Com4 has been shown by ITC to interact with negatively charged membranes with micromolar affinity [45]. Hence, there should be little competition from serum albumin to sequester the peptides away from the bacterial cells. Another possible consequence of the weak interactions could be increased trafficking of the peptide through the circulatory system, given the transporting role of albumin for many molecules such as hormones and fatty acids. In another study, trimeric peptides that interact with albumin at a 1∶1 ratio and a micromolar *K_D_* were found to be compromised in their antimicrobial activity due to the addition of BSA to the *in vitro* assays [33].

There are many degradative enzymes present in the human circulatory system. Serum proteases have regulatory roles for enzymes and other proteins involved in the immune system and blood clotting. As well, proteolytic activity serves to limit the lifetimes of blood proteins and peptides such as antibodies and hormones. The issue of peptide degradation will grow in importance as more peptide candidates with promising levels of antimicrobial activity enter the clinical stages of drug development. For example, recent studies have used fusogenic liposomes containing the RRWQWR peptide (Lfc1) to kill cancer cells [53]. The modified peptides discussed here could have improved anticancer activity and perhaps be delivered more directly.

In this study, the effects of several simple modifications were explored for short Trp- and Arg-rich peptides on their proteolytic stability. The serum degradation profiles of the peptides also give a first indication as to their resistance against secreted bacterial proteases. However, the proteolytic stabilities determined here do not have a clear correlation with biological activities, which emphasizes that the modifications also affect the peptides' antimicrobial mechanisms. The principles revealed here regarding end capping, peptide cyclization and peptide length will help guide the design of optimal antimicrobial, and possibly anticancer, candidates for further pharmaceutical development.

## Materials and Methods

All peptides were synthesized using standard 9-fluorenylmethyoxycarbonyl (Fmoc) chemistry and purified by HPLC to >95% purity. They were obtained from Anaspec, Inc (San Jose, CA) except for Com7, which was purchased from 21^st^ Century Biochemicals (Marlboro, MA). Male human serum (H-4522), >96% pure BSA (Lot 128H0656), and >99% pure HSA (Lot 97H7604) were obtained from Sigma-Aldrich. The concentrations of the peptides and the albumins were calculated by UV absorption at 280 nm using extinction coefficients determined by ProtParam [54].

### Serum stability assay

Peptide stabilities were assayed in diluted serum as previously described [55]. 25% human male serum was centrifuged at 13,000 rpm for 10 min to remove lipids and the supernatant was collected and incubated at 37°C for at least 15 min. The assay was initiated upon the addition of the test peptide to the serum for a final peptide concentration of 5 µM. 200 µL aliquots of the incubations were taken for the following time points: 0, 30, 75, 120, 240, 390 and 540 min. The aliquots were mixed with 40 µL of 15% trichloroacetic acid (TCA) and incubated at 4°C for at least 15 min to precipitate serum proteins. In the case of Com5 and Com6, 5 µL of 1 M NaOH was supplemented to the TCA to prevent peptide precipitation. The supernatant was collected for each sample after centrifugation at 13,000 rpm for 10 min and stored at −20°C. These assays were performed in triplicate.

Reverse phase high performance liquid chromatography (RP-HPLC) analysis was performed with an AKTA Purifier from Amersham Biosciences (Westborough, MA) using an uRPC C2/C18 ST 4.6/100 column from Pharmacia Biotech (SanJose, CA) of the following dimensions: bed length 100 mm, i.d. 4.6 mm, 120Å. Individually thawed samples were injected through a 100 µL loop, after which a linear gradient was run from 100% solution A (0.05% trifluoroacetic acid (TFA) in filtered water) to 100% solution B (0.045% TFA in 90% acetonitrile) with a flow rate at 0.5 mL/min. The relative concentrations of the remaining soluble peptides were analyzed by the integration of the absorbance at 215 nm as a function of retention time using the Analysis module of the Unicorn software package.

### MALDI-TOF mass spectrometry

Samples from the serum stability assay of each peptide after 75 min incubation time were analyzed for peptide fragments by MALDI-TOF mass spectrometry with a Voyager DE-STR (Applied Biosystems) using the two layer method. Briefly, a sinapinic acid (Sigma S8313) matrix solution of 20 mg/mL in acetone:methanol (80∶20) was placed on a MALDI target (0.5 µL) as a seed layer and allowed to dry. A second matrix solution of 10 mg/mL formic acid: isopropanol: HPLC water (1∶2∶3) was mixed with equal amonts of sample (diluted to ∼1–5 µM in formic acid: isopropanol: HPLC water solution) and 0.5 µL of mixture was placed on top of seed layer and allowed to dry. The MALDI-TOF was operated in the linear mode and calibrated externally using a protein mixture of insulin, myoglobin and cytochrome c.

### Isothermal titration calorimetry

ITC experiments were performed at 25°C on a Microcal VP-ITC microcalorimeter with data analysis performed with the Microcal Origin software. The sample cell contained 0.1 mM of either BSA or HSA dissolved in 20 mM HEPES, 100 mM NaCl, pH 7.9. The syringe was loaded with 2 mM test peptide in the same buffer, and each titration point resulted from 5 µL injections following an initial 2 µL injection. Separate control experiments were also performed to account for heats of dilution/mixing.

### Bactericidal assays

The peptides were tested for microbicidal activities against *Bacillus subtilis* ATCC6633, *Escherichia coli* ML35, and *Staphylococcus aureus* 42D as previously described [56]. Suspensions of logarithmically growing test bacteria were incubated on a microtiter plate at a concentration of 10^5^ colony-forming units/mL with various peptide dilutions. The medium was 100 µL of 10 mM phosphate buffer pH 7.0 supplemented with 1% (v/v) of tryptic soy broth (TSB). For later assays, human male serum was also added to a final concentration of 25%. 10 µL aliquots were collected at time 0 h and after 2 h of incubation on a rotary shaker (300 rpm) at 37°C. These were plated on blood agar plates and incubated overnight at 37°C. The Lethal Dose concentration (LD_99.9_) was determined, corresponding to the concentration killing >99.9% of the inoculum at 2 h. These experiments were performed in duplicate.

For the testing of the peptides' activities in different conditions, e.g. with serum, we use the same cut-off for activity as defined for Minimum Bactericidal Concentration (MBC), i.e. a 1000-fold reduction of the inoculum. However, we designate this peptide concentration with the more general term LD_99.9_ which can be applied to the specified conditions of the experiments performed here.

### Hemolysis assays

The peptides were also tested for hemolytic activity in assays using red blood cells from heparinized human blood as previously described [57]. Fresh human blood was centrifuged at 1500 rpm for 10 min as 4°C and washed with phosphate buffered saline, pH 7.3. Red blood cells were diluted to 1% and incubated with various peptide dilutions for 1 h at 37°C. These incubations were then centrifuged at 4000 rpm for 5 min and the supernatant was collected for absorbance measurements at a wavelength of 540 nm. Controls for 0% and 100% hemolysis were determined, with the latter condition defined by lysis by 1% Triton X-100. Effective Concentrations for 50% hemolysis, EC_50_, were determined from the resulting dose-response profiles.

## Supporting Information

Figure S1 Example ITC isotherms of 2 mM peptide into 0.1 mM serum albumin at 25°C, fitted using two-site modeling. Profiles of A) Lfc8 titrated into HSA (strongest KD  = 0.15 mM), and B) Com7 titrated into BSA (strongest KD  = 0.21 mM).
(0.46 MB TIF)
