# Supplementary figures and images for: Serum Stabilities of Short Tryptophan- and Arginine-Rich Antimicrobial Peptide Analogs
Source: PLoS One. 2010 Sep 10;5(9):e12684. doi: 10.1371/journal.pone.0012684 (PMC2937036; doi:10.1371/journal.pone.0012684)

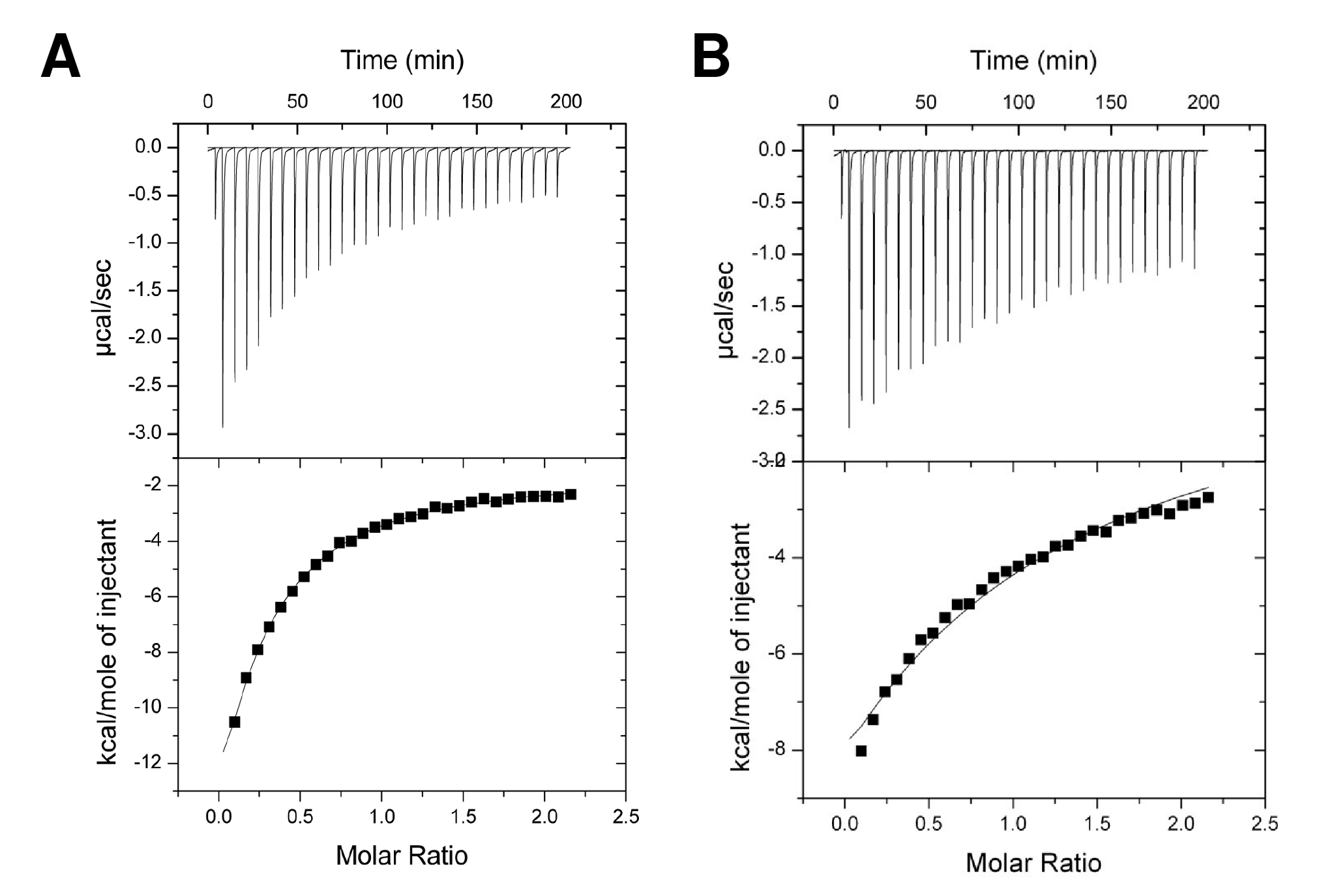

Supplement: Figure S1 — Example ITC isotherms of 2 mM peptide into 0.1 mM serum albumin at 25°C, fitted using two-site modeling. Profiles of A) Lfc8 titrated into HSA (strongest KD = 0.15 mM), and B) Com7 titrated into BSA (strongest KD = 0.21 mM). (0.46 MB TIF) [file pone.0012684.s001.tif]
